# Management of a Pulmonary Sequestration With a Large Aberrant Artery Using a Hybrid Approach: Thoracic Endovascular Aortic Repair Followed by Video‐Assisted Thoracoscopic Lobectomy

**DOI:** 10.1155/cris/9974576

**Published:** 2026-02-13

**Authors:** Evangelos Koliakos, Philippe Charbonneau, Moishe Liberman, Basil Nasir, Andrei-Bogdan Gorgos, Pasquale Ferraro

**Affiliations:** ^1^ Division of Thoracic Surgery, University of Montreal Hospital Center (CHUM), Montreal, Quebec, Canada; ^2^ Division of Vascular Surgery, University of Montreal Hospital Center (CHUM), Montreal, Quebec, Canada; ^3^ Division of Radiology, University of Montreal Hospital Center (CHUM), Montreal, Quebec, Canada

## Abstract

Pulmonary sequestration is a rare congenital malformation of the lower respiratory tract, characterized by nonfunctioning lung tissue without tracheobronchial communication and receiving its blood supply from the systemic circulation. It can present as either intralobar or extralobar sequestration, with intralobar being more common. Surgical resection remains the treatment of choice, although preoperative strategies such as endovascular occlusion can reduce the risk of intraoperative complications. We report the case of a 52‐year‐old woman with a history of recurrent pulmonary infections who was referred for evaluation of an incidentally discovered cystic lesion in the left lower lobe following a transient episode of amaurosis fugax. Chest CT revealed a 5.3 cm pulmonary sequestration supplied by a large aberrant artery (AA) originating from the descending aorta. A two‐stage hybrid procedure was performed, starting with thoracic endovascular aortic repair (TEVAR) to exclude the AA, followed by video‐assisted thoracoscopic surgery (VATS) left lower lobectomy. The patient had an uneventful recovery and was discharged postoperatively. TEVAR was employed as a preoperative strategy to minimize the bleeding risks associated with the large AA during minimally invasive lobectomy. A hybrid two‐stage approach using TEVAR followed by VATS is a safe and effective method for managing intralobar pulmonary sequestration with large aberrant vessels. Careful preoperative planning and a multidisciplinary approach are essential for optimal outcomes.

## 1. Introduction

First reported by Peyce in 1946 [1], pulmonary sequestration is a rare congenital malformation of the lower respiratory tract accounting for less than 6.5% of all congenital pulmonary malformations [2–4].

The definition of a pulmonary sequestration is a nonfunctioning lung tissue without tracheobronchial communication that receives blood supply from the systemic circulation rather than the pulmonary circulation [2].

Two types of pulmonary sequestration exist. The intralobar type shares the visceral pleura with the rest of the normal lung parenchyma, while the extralobar type is separated from the normal lung tissue by its inherent pleura [5]. Intralobar sequestrations account approximately for 75% of cases, and their localization is most commonly (98%) in the lower lobes [3].

In most cases, pulmonary sequestration supplying arteries originate from the descending aorta, while there have been reports of the aberrant artery (AA) branching from the abdominal aorta, the splenic or left gastric artery [6].

Pulmonary sequestration can be completely asymptomatic and is therefore most frequently diagnosed in adult patients, classically following recurrent pulmonary infections [6]. However, cases of massive hemoptysis have been described [7].

The high likelihood of recurrent infection, the potential need of a larger resection in chronic sequestrations as well as the elevated risk of massive bleeding and hemoptysis mandate an aggressive management of this rare congenital disease [8]. Surgical resection with concomitant isolation of the aberrant feeding arteries is the treatment of choice for pulmonary sequestration [6, 8–10].

Here, we present the case of an intralobar pulmonary sequestration supplied by an AA branching from the descending aorta, managed with a hybrid surgical two‐stage procedure: thoracic endovascular aortic repair (TEVAR) and subsequent video‐assisted thoracoscopic surgery (VATS) left lower lobectomy.

## 2. Case Presentation

This is the case of a 52‐year‐old female patient who was referred to our thoracic surgery clinic for evaluation after the discovery of a large cystic lesion of the inferior left lobe.

The patient had no medical history other than an episode of amaurosis fugax, which led to a chest CT scan. Upon further anamnesis, she mentioned a history of recurrent pulmonary infections during her younger age. The patient was completely asymptomatic at the time of consultation.

**Figure 1:**
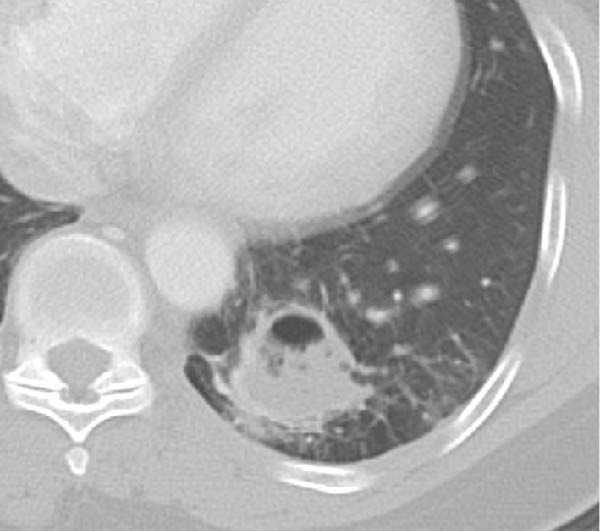
Cystic lesion of the left lower lobe measuring 2.4 × 3 × 5.3 cm.

A chest CT revealed a cystic lesion of the left lower lobe measuring 2.4 × 3 × 5.3 cm (Figure 1). A large aberrant nourishing vessel with a diameter of 17 mm originating directly from the descending aorta was identified with a venous drainage mainly to the hemiazygos vein and left inferior pulmonary vein (Figure 2).

Figure 2(A) Cystic lesion with a large aberrant artery of 17 mm of diameter originating directly from the descending aorta. (B) Red asterisk demonstrating the aberrant artery, blue asterisk demonstrating the venous drainage in the hemiazygos vein. (C) 3D reconstruction of the descending aorta.

**Figure:**
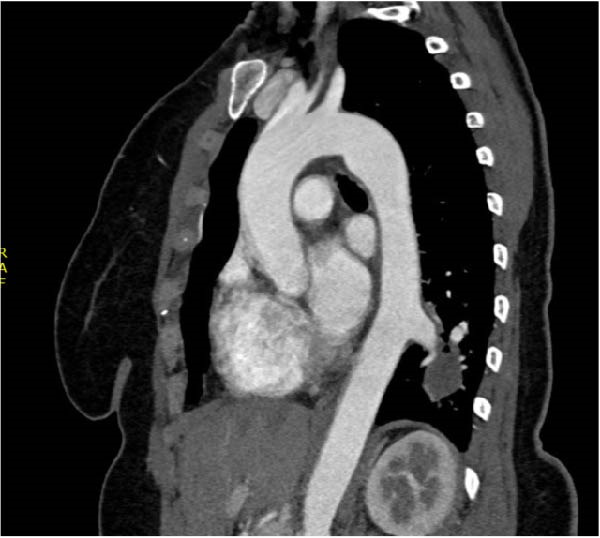
(A)

**Figure:**
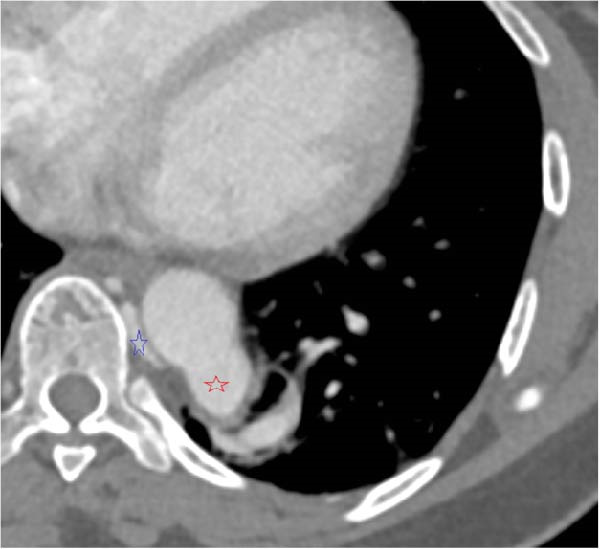
(B)

**Figure:**
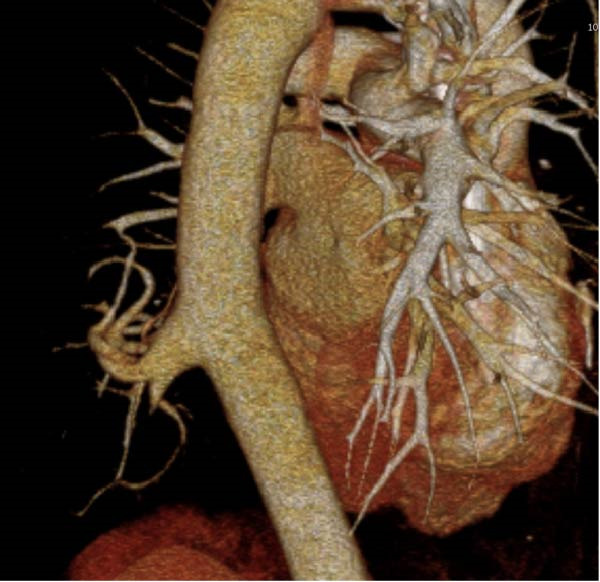
(C)

Bronchoscopy findings were normal, while the bronchoalveolar lavage didn’t show any malignant cells, and microbiological examination was negative.

After discussion with our vascular surgery colleagues, we decided to proceed with a TEVAR to exclude the origin of the AA, followed by a VATS left lower lobectomy in a two‐stage approach.

The endovascular procedure was performed under general anesthesia in a hybrid operating room. A percutaneous femoral access was achieved, and a bolus of 6000 units of heparin was administered. Fusion imaging guidance was used to deploy a Cook (Bloomington, Indiana) Alpha Thoracic Endograft of 26 mm × 105 mm, a few centimeters above the celiac trunk. An aortic sealing zone of more than 3 cm in length was obtained on each side of the origin of the AA. A molding of the endograft with a compliant endovascular balloon allowed a better apposition to the aortic wall. The final angiogram showed a late type 4 endoleak (Figure 3).

Figure 3(A) Initial aortogram. (B) Final aortogram following TEVAR procedure. Red star: succesful AA exclusion following the placement of thoracic aorta endograft, Cook (Bloomington, Indiana) Alpha Thoracic Endograft of 26 mm × 105 mm.

**Figure:**
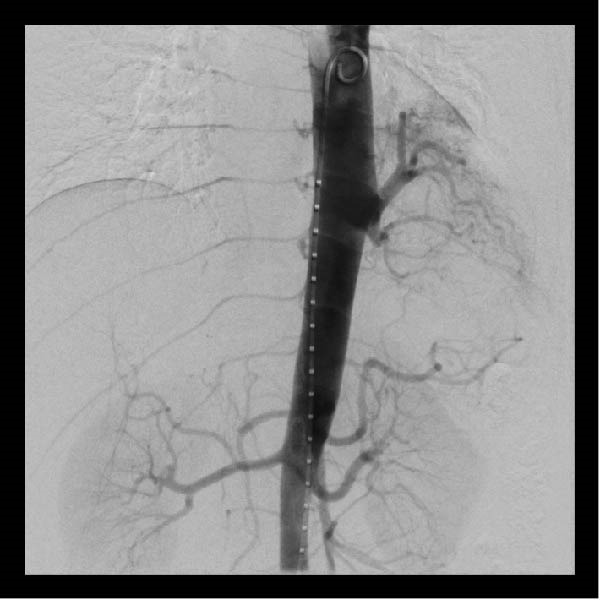
(A)

**Figure:**
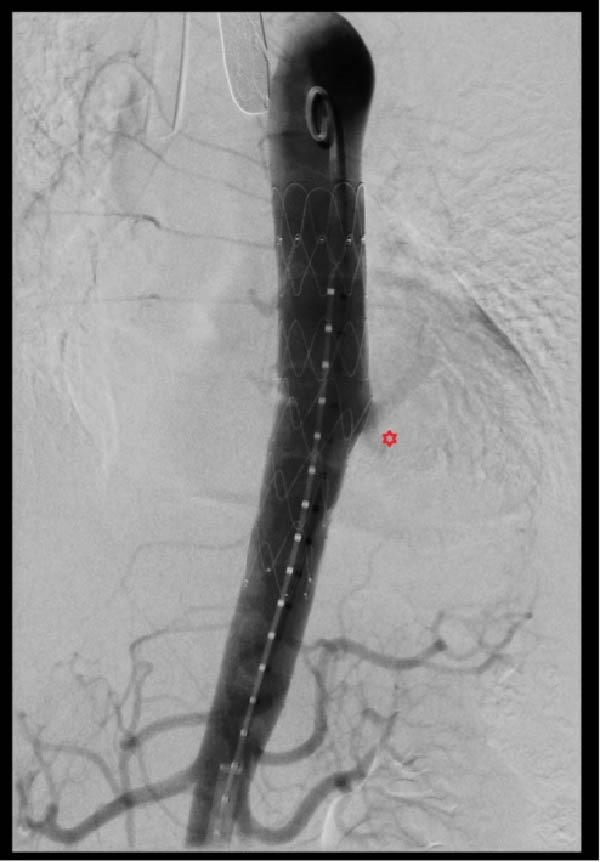
(B)

**Figure 4:**
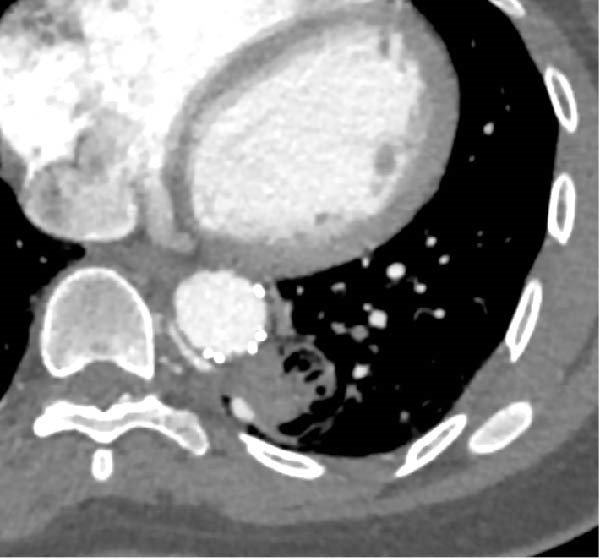
Thoracic aorta with endograft successfully excluding the nourishing vessel with slight signs of retrograde flow probably through the azygos vein.

A chest angio‐CT was conducted on POD 1 following the endovascular procedure. The thoracic endograft successfully excluded the AA, while slight signs of retrograde flow were detected, probably due to backflow through the azygos vein system and pulmonary artery (Figure 4).

Recovery was uneventful, and the patient was discharged 72 h following the endovascular procedure with a prescription of 100 mg of acetylsalicylic acid per day.

**Figure 5:**
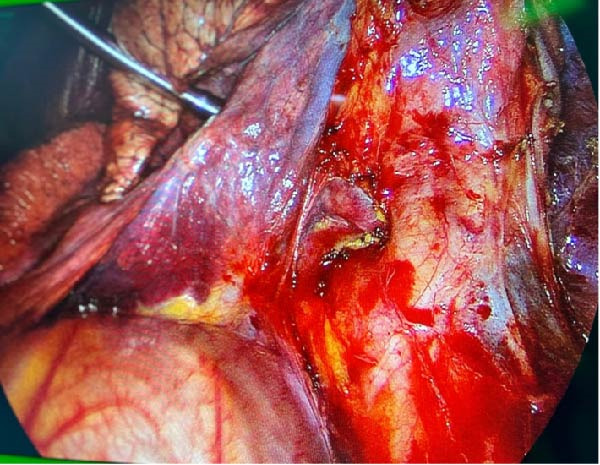
Giant nourishing artery of the sequestration branching directly from the descending aorta as identified during VATS after dissection of the inferior pulmonary ligament. Visualization of the inferior pulmonary vein.

About 1 month later, the patient was readmitted for the programmed lobectomy. An anterior three‐port VATS was carried out, and after some minor adhesiolysis, the supplying artery of the sequestration was identified branching directly from the descending aorta (Figure 5). A careful dissection of the vessel allowed a loop placement and ligation using an Ethicon Echelon Flex 45 mm Vascular Stapler (Figure 6). A left lower lobectomy was carried out in a standard fissureless approach. The chest tube was weaned on POD1, and the patient was discharged the following morning.

Figure 6(A) Ligation of the aberrant artery using a stapling device. (B) Stapler line on the descending aorta.

**Figure:**
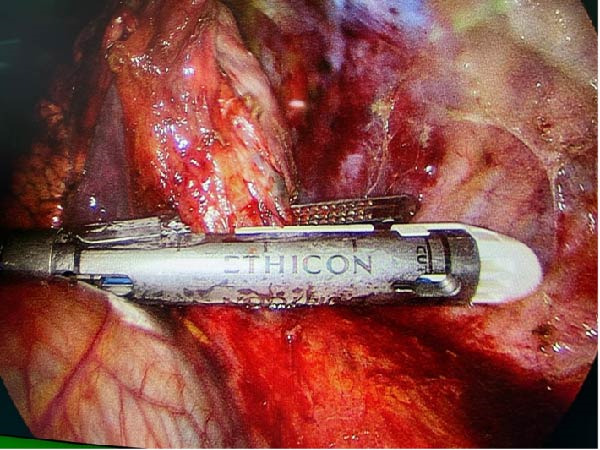
(A)

**Figure:**
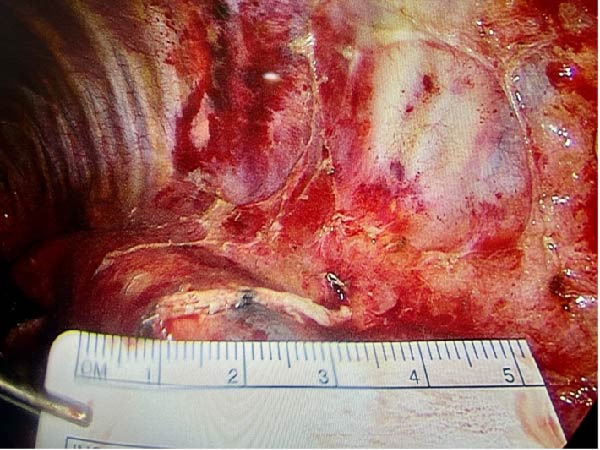
(B)

The final pathological examination demonstrated a type II pulmonary malformation of the left lower lobe, consistent with an intralobar pulmonary sequestration, associated with degenerative changes of the resected systemic arterial segment.

The patient had an uneventful recovery and continues to do well several months following surgery.

## 3. Discussion

Surgical resection of pulmonary sequestration is recommended in order to eliminate the risk of infection and bleeding, while it allows for confirmation of diagnosis through the final pathological report [6, 8–12].

Concerning the extent of resection, intralobar pulmonary sequestration is usually managed by lobectomy, while sublobar resections can also be performed for smaller sequestrations localized peripherally [8]. The conventional surgical approach for resection by a posterolateral thoracotomy has gradually given way to minimally invasive approaches such as VATS and robot‐assisted thoracic surgery (RATS), which have been shown to be equally effective and safe when performed by experienced surgical teams [9]. Nevertheless, no treatment guidelines have been established to date.

Endovascular occlusion of the arterial supply can be used as a useful preoperative approach in order to reduce blood flow towards the sequestration and minimize the risk of intraoperative bleeding, especially in the setting of minimally invasive surgery [8]. TEVAR can be useful when the origin of the feeding vessel, which is providing blood supply to the sequestration, has a large diameter [10, 11]. Although no clear size threshold has been established in the current literature, several authors report that AAs with a large caliber, short intrathoracic course, or aneurysmal morphology may represent a higher risk of intraoperative bleeding when managed surgically, particularly during minimally invasive approaches [9–11]. In clinical practice, arteries exceeding 10 mm in diameter may be considered large, although no specific diameter cutoff has been formally validated from which preoperative endovascular exclusion can be recommended to depressurize the vessel and facilitate safer surgical ligation. Embolization of the nourishing vessels as a definitive treatment of pulmonary sequestrations has been reported in the literature; however, concerns about long‐term recurrence of infection, incomplete exclusion of the sequestration, and lack of pathological diagnosis represent the main inconveniences of this therapeutic option as a definitive treatment [10, 13].

In their study, Zhang et al. [12] retrospectively studied 28 patients receiving surgery or endovascular treatment for management of pulmonary sequestration. They concluded that a solely endovascular treatment could be considered for small‐sized pulmonary sequestrations under 3 cm in diameter.

In our patient’s case, we proceeded with a two‐stage hybrid endovascular procedure associated with a minimally invasive surgical approach, allowing for a safe and definitive management of this rare congenital disease. The preoperative planning with the use of high‐resolution angio‐CT revealed an intralobar pulmonary sequestration of 5 cm with a large AA measuring 17 mm in diameter (Figures 1 and 2), which represented a therapeutic challenge.

Nourishing vessels of pulmonary sequestrations carry blood under systemic pressure and are known to have fragile and thin walls due to recurrent infectious processes [10]. Liu et al. [9] have reported that iatrogenic vascular injury of the AA of pulmonary sequestrations is the most common complication and reason for emergency conversion to thoracotomy when performing a resection by VATS. Similarly, Zhang et al. [12] reported that even in open surgery, management of an intraoperative bleeding of the aberrant vessel can be challenging, with two patients presenting a blood loss greater than 1000 mL in their case series.

In our patient’s case, given the size of the AA, we considered that its exclusion with an endograft would be the safest approach. The AA was large from its aortic origin and rapidly divided into multiple branches. Ligation of a short, large, and fragile artery, which is still pulsating, has a considerable risk of tear and subsequent uncontrolled hemorrhage. TEVAR as a first stage has the advantage of depressurizing the AA and allows a safer ligation, while decreasing the long‐term potential risk of rupture from an aortic stump degeneration. TEVAR has a risk of spinal cord ischemia, although the probability is low when the aortic length coverage is less than 150 mm [14].

The placement of the endograft and subsequent exclusion of the aberrant vessel allowed us to perform the resection of the pulmonary sequestration through VATS, which has become more and more popular for surgical management of this pathology during the recent years [6, 9, 11].

## 4. Conclusion

We presented a safe and minimally invasive two‐stage hybrid procedure for the management of an intralobar pulmonary sequestration with a large aberrant vessel. We highlight herein the importance of careful and meticulous preoperative planning as well as the significance of a multidisciplinary approach, allowing for optimal results while using minimally invasive techniques.

## Funding

No funding was received for this manuscript.

## Consent

Written informed consent has been obtained from the patient.

## Conflicts of Interest

The authors declare no conflicts of interest.

## Data Availability

Data sharing is not applicable to this article, as no new data were created or analyzed in this study.
